# Research on the Influence of Recycled Fine Powder on Chloride Ion Erosion of Concrete in Different Chloride Salt Environments

**DOI:** 10.3390/ma18092018

**Published:** 2025-04-29

**Authors:** Lijun Chen, Gang Zhao, Ying Li

**Affiliations:** 1 School of Civil Engineering and Water Resources, Qinghai University, Xining 810016, China; 2 Key Laboratory of Material and Engineering Safety for Building Energy Conservation in Qinghai Province, Xining 810016, China

**Keywords:** chloride ion erosion, recycled fine powder, sodium chloride solution, Salt Lake brine solution, free chloride ion diffusion coefficient

## Abstract

The Qinghai–Tibet Plateau features a high-altitude, cold, and arid climate, with harsh environmental conditions. It is also one of the regions in China where chloride-rich salt lakes are abundant. These circumstances pose significant challenges to the durability of concrete. This study explored the impact of recycled fine powders (RFP) on the resistance of concrete to chloride ion erosion. To evaluate this, a 3.5% sodium chloride solution and Qarhan Salt Lake brine were employed as erosion media. The depth and concentration of chloride ion penetration, the free chloride ion diffusion coefficient (D_f_), and the microstructure of the concrete were measured. The results demonstrated that when the replacement rate of RFP was 20%, the concrete displayed excellent resistance to chloride ion erosion in both the sodium chloride solution and the Salt Lake brine. XRD analysis and SEM images revealed that the addition of RFP enabled the concrete to bind more Cl^−^ to form Friedel’s salt, which filled the pores of the concrete and reduced the diffusion of Cl^−^ within the concrete. Moreover, as the soaking time extended continuously, the erosion and damage effects of the Salt Lake brine solution on the concrete were more severe than those of the sodium chloride solution.

## 1. Introduction

In recent years, urbanization and construction in China have been accelerating continuously. The environmental pollution resulting from construction waste, generated during the demolition of existing buildings and the renovation of old buildings, should not be underestimated. Recycling and utilizing construction waste can not only resolve the current situation of cities being besieged by garbage, but also effectively alleviate the shortage of natural sand and gravel materials in China at present. Moreover, it can reduce the consumption of cement clinker, which is of great significance for cutting greenhouse gas emissions and achieving the carbon-reduction targets of the cement and concrete industry [1,2,3,4,5].

Previous studies have shown that incorporating mineral admixtures such as fly ash into concrete can effectively improve the internal pore structure of concrete and has a positive effect on enhancing its ability to resist the influence of the external environment. Therefore, mineral admixtures such as fly ash, silica fume, etc., have been widely used in high-performance concrete, and in many places, there is even a situation where mineral admixtures are in short supply. Therefore, it is imperative to develop new mineral admixtures for the production of concrete [6]. In the process of producing recycled aggregates from construction waste, a large amount of fine powder with a particle size less than 0.075 mm, namely recycled fine powder (RFP), is often generated. Singh [7] found that using RFP can lead to stable, low-carbon concrete and contribute to a sustainable automated construction industry. A large number of studies [8,9,10,11] have shown that the main components of RFP are CaO, SiO_2_, etc., which have hydration activity close to that of fly ash. When used to replace some of the cement in concrete, it can play the role of micro-aggregate filling and secondary hydration. Under an appropriate substitution rate, it can improve the durability of concrete, such as frost resistance and resistance to chloride ion erosion. Xiao et al. [12] showed that the carbonation depth and carbon absorption of concrete gradually increased with the increase in the replacement rate of RFP. Mao et al. [13] showed that controlling the fineness and dosage of RFP appropriately can effectively improve the resistance of concrete to chloride ion penetration. Ma [14] confirmed that the micro-aggregate filling effect and volcanic ash reaction of RFP can improve the pore structure, and also helps to form C-S-H with low Ca/(Si+Al) and Ca/Si, which can enhance the resistance of concrete to chloride erosion. Bogas et al. [15] indicated that for up to 15% replacement with RFP, the concrete durability was not significantly affected. However, Sun et al. [16] showed that RFP content has negative effects and positive effects on the chloride penetration behavior. Bian et al. [17] found that when the RFP content was less than 50%, the corrosion resistance coefficient of the compressive strength of the mortar was 0.84–1.05 after 90 days of sulfate attack. In the above studies, it was shown that the addition of RFP can refine the pore structure in cement-based material and is beneficial for improving the durability of concrete at a certain substitution rate.

The Qinghai region is located in the Qinghai–Tibet Plateau, with a cold and arid climate and a harsh environment. It is one of the regions where salt lakes are widely distributed in China. The brine environment of salt lakes contains a large number of erosive ions, such as Cl^−^ and SO4^2−^. The interaction of these ions changes the transport law of chloride ions and affects the capacity of concrete to resist chloride ion erosion, posing a serious threat to the durability of concrete. Ordinary concrete will undergo corrosion cracking within 2 to 3 years of service [18,19,20]. Numerous previous studies [21,22] have indicated that incorporating mineral admixtures, such as fly ash and slag, into concrete exerts a positive effect on enhancing its resistance to chloride and sulfate corrosion. However, in comparison with fly ash and silica fume, research on the effect of RFP on the durability of concrete, such as its resistance to chloride ion erosion and sulfate erosion, is relatively scarce. This situation, to a certain extent, restricts its application scope, especially when used in the Qinghai Plateau region.

Therefore, in the present study, RFP was employed to substitute a portion of cement at replacement rates of 0%, 10%, 20%, 30%, and 40% in order to produce recycled concrete. Subsequently, the resultant concrete specimens were immersed in the Qinghai Cha’erhan Salt Lake solution and a 3.5% sodium chloride solution. The soaking period was 240 days, and the spreading performance of chloride ions in concrete was tested in a cycle with an interval of 30 days to investigate the effect of RFP on the resistance of concrete to chloride ion erosion. In addition, the microstructure of the recycled concrete was explored to investigate the effect mechanism of RFP on the recycled concrete. The results of this study can provide a theoretical basis and practical guidance for reducing the potential threat of the salt lake environment to the durability and safety of recycled concrete projects.

## 2. Materials and Methods

### 2.1. Raw Materials

P.O 42.5 Ordinary Portland cement (Jinyuan, Huzhu, China) was used in this study. Recycled fine powder (RFP) was produced by crushing a waste concrete beam with a jaw crusher (XJKEP-3, Xinjinke, Taiyuan, China), pulverizing this material with a PM2L planetary ball mill (PM2L, Zhuode, Shaoxing, China) for 30 min, and screening the powder through a 0.075 mm sieve. The chemical composition and physical properties of the cement and the RFP are shown in Table 1 and Table 2.

**Figure 1:**
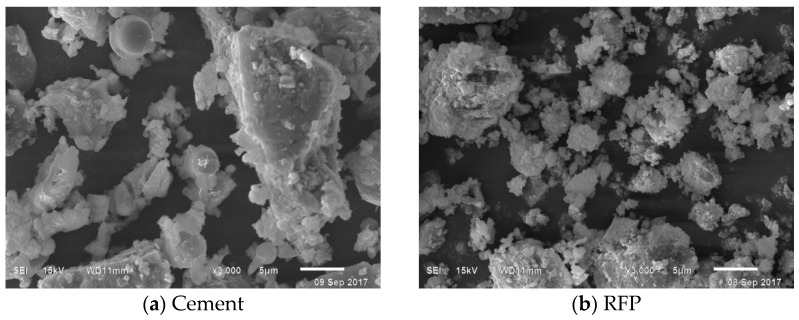
SEM diagram of cement and RFP.

As shown in Figure 1, compared to cement, the surface of the RFP is rough, with the particles mostly being irregular blocks and flakes, and the texture is loose. This is mainly due to the secondary mechanical crushing process, which caused a large number of micro cracks and mutual adhesion to be generated in the RFP.

The particle size of the coarse aggregate ranged from 4.75 mm to 20 mm. The fine aggregate was river sand with a particle size of 0.15 mm to 5 mm, and its fineness modulus was 2.80. The basic physical properties of the coarse aggregates and fine aggregates are shown in Table 3.

The Salt Lake brine used in the experiment was sourced from the Qarhan Salt Lake in Qinghai Province. The ion concentrations in the Salt Lake brine were detected, and the test results are shown in Table 4. The main anions in the Salt Lake brine are Cl^−^ and SO_4_^2−^, and the main cations are Mg^2^⁺, Na^+^, and K^+^. Among them, the anion with the highest concentration is Cl^−^, with a concentration of 201 g/L; the cation with the highest concentration is K^+^, with a concentration of 79 g/L. The sodium chloride solution used in this experiment was obtained by mixing sodium chloride crystal powder with tap water from the laboratory. The concentration of chloride ions in this solution was 3.5%.

### 2.2. Mix Proportion and Specimen Preparation

#### 2.2.1. Mix Proportion

Four mix proportions, along with the cement dosages by weight (10%, 20%, 30% and 40%) replaced by RFP, are given in Table 5. Concrete without added RFP was used as the control group. The W/C ratio was kept constant at 0.45. Methacrylate polycarboxylate superplasticizer (0.5% of cement mass) was added to maintain equal flow ability between the test groups.

#### 2.2.2. Specimen Making

According to the “Standard for Test Methods of Long-Term Performance and Durability of Ordinary Concrete” (GB/T 50082-2009), 100 mm × 100 mm × 100 mm cube specimens were fabricated in accordance with the mix proportion design method in Table 5. The cast specimens were placed in a constant-temperature curing room at 20 °C for 24 h and then demolded and placed in a standard curing room (20 ± 2 °C, relative humidity above 95%) for curing. After 28 days of curing, five surfaces of the specimens were coated and sealed with epoxy resin, and after drying for 1 day in air, one surface was immersed in 3.5% sodium chloride solution or Salt Lake brine, and the salt solution was replaced every 7 days.

### 2.3. Test Methods

The methods for testing the chloride ion erosion resistance of concrete mainly include the natural diffusion method [23], the electric flux method [24], the RCM method [25], etc. Among them, the natural diffusion method is the most direct one and is also more in line with the actual engineering situation. In this method, after the concrete specimens are cured to the specified age, they are all immersed in a NaCl solution of a certain concentration. After reaching the specified immersion period, concrete powder at different depths is obtained by means of drilling sampling. Then, the chloride ion concentration in the specimens is measured using chemical methods. Subsequently, the chloride ion diffusion coefficient D is fitted according to Fick’s second law [23]. Therefore, this paper uses the natural diffusion method to evaluate the performance of recycled fine powder concrete in resisting Cl^−^ transport.

**Figure 2:**
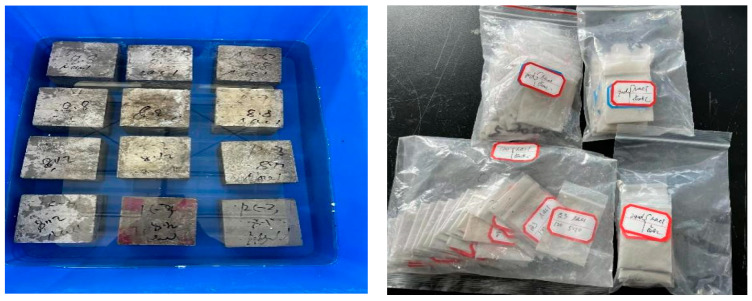
Immersion of test specimens and sampling.

In this study, in accordance with relevant standards including the “Code for Durability Design of Concrete Structures” (GB/T 50476-2008) and the “Test Code for Concrete in Water Transport Engineering” (JTJ 270-98), the cured concrete specimens were subjected to natural immersion in a salt solution. Subsequently, the water-soluble chloride ion content within the concrete was expeditiously ascertained through the ion-selective electrode (ISE) method. First, the drilling sampling method was used to collect sample powder at different depths in concrete test blocks. Specifically, after the above specimens were soaked for 28 d, 60 d, 90 d, 120 d, and 240 d, two of them were taken out and naturally dried for 1 day. Powder samples were taken at 5 points evenly on the surface without epoxy resin with a hammer drill with a diameter of 8 mm. Samples were taken from the position of 2.5 mm and at a depth of 5 mm until a depth of 25 mm. The sample was screened with a standard screen with a diameter of 0.63 mm, and then the sifted powder was placed in the oven at 105 ± 5 °C for 2 h, and the package number was set up for use after drying. The soaking and sampling processes are shown in Figure 2.

The selected sample was divided into two parts and dissolved in ultra-pure water. The Cl^−^ content rapid tester (SSWY-810, Beijing Shengshi Weiye Technology Co., Ltd., Beijing, China) was used for the electrode tests to determine the mass percentage of Cl^−^ content relative to the mass of concrete (C_f_) in the sample. The test time of each test block was 120 s. Before each test, standard liquid was used to calibrate the instrument. An electronic balance with an accuracy of 0.0001 g was used to weigh an appropriate amount of concrete powder sample. Then, the sample was placed in a small 100 mL beaker, an appropriate amount of distilled water was added, and the mixture was stirred using a magnetic stirrer for 30 min. The chloride-ion-selective electrode activated in a 0.1 mol/L NaCl solution and the assembled calomel electrode were inserted into the beaker, and the chloride ion concentration and free Cl^−^ ion concentration C_f_ were observed when the reading was stable [26].

## 3. Results and Analysis

### 3.1. Distribution of Cl^−^ Ion of Concrete in Sodium Chloride Solution

**Figure 3:**
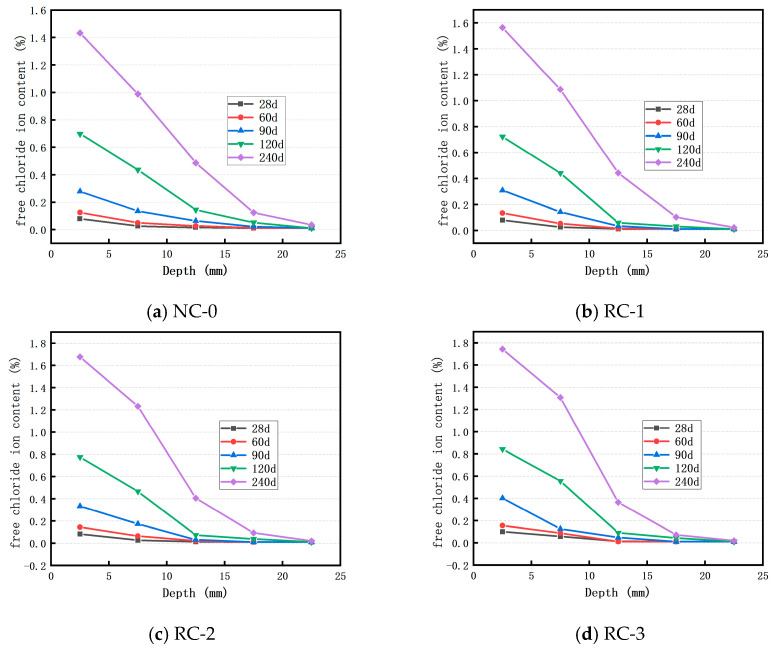

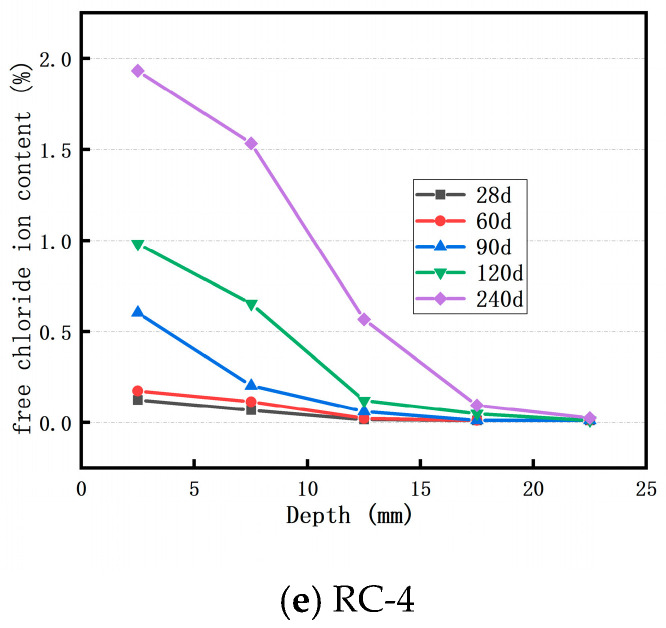
Distribution of free Cl^−^ ion content of concrete in sodium chloride solution.

Figure 3a–e depict the distribution of the free chloride ion content at varying depths within the control concrete and the concrete incorporating 10%, 20%, 30%, and 40% RFP subsequent to immersion in a 3.5% sodium chloride solution for 28, 60, 90, 120, and 240 days, respectively. Apparently, for all concretes, the Cl^−^ ion concentration at the same depth increases as the soaking time lengthens. Under identical soaking time conditions and at the same depth position, the concentration of free Cl^−^ ions also rises with an increasing RFP substitution rate. When the RFP substitution rate reaches 40%, the Cl^−^ ion concentration at a depth of 2.5 mm after 240 days of soaking is the highest, reaching 1.9326%, which represents a 34.94% increase compared to that of the ordinary concrete in the control group. This is because with the increase in RFP replacement rate, the cement content in concrete decreases, resulting in fewer hydration products. Insufficient hydration products within the concrete fail to adequately fill the pores, thereby facilitating the relatively easy diffusion of Cl^−^ ions. Within the same soaking timeframe, as the diffusion depth increases, the corresponding free Cl^−^ ion concentration decreases. The free Cl^−^ ion concentration up to a surface depth of 7.5 mm is higher than that in the control group, while the Cl^−^ ion concentration beyond a surface depth of 12.5 mm is lower than that in the control group. This indicates that the diffusion of Cl^−^ ions in the concrete is relatively impeded. The reason for this lies in the fact that RFP exhibits a certain adsorption effect on Cl^−^ ions, and the pores in the concrete with RFP are larger than those in the control concrete, resulting in the accumulation of Cl^−^ ions on the surface of the RFP-incorporated concrete [27]. However, during the concrete soaking process, some unhydrated RFP will undergo further hydration and fill some of the pores. Simultaneously, its rough surface structure can enhance the resistance to Cl^−^ ion migration, rendering it difficult for Cl^−^ ions to migrate within the concrete. Overall, it can be observed that in RFP-containing concrete, the Cl^−^ ion concentration measured at the same position increases with the extension of soaking time, gradually decreases with the increase in depth, and eventually stabilizes, in accordance with the Cl^−^ ion diffusion law in concrete blended with ceramic powder, slag, and fly ash [28,29,30].

**Figure 4:**
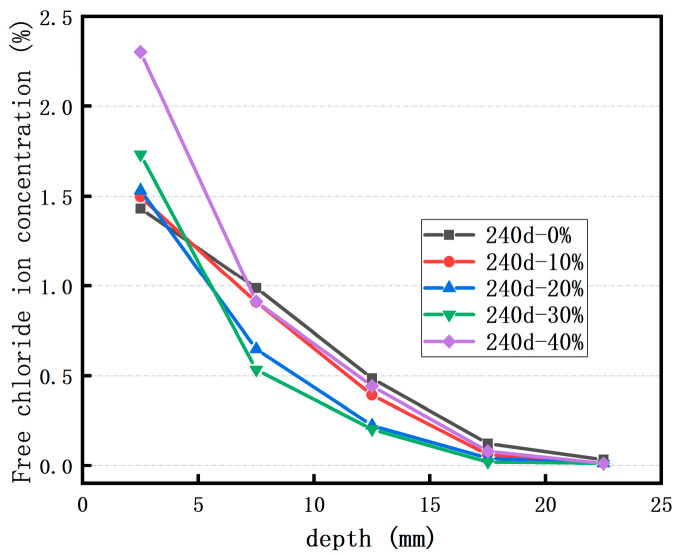
Effect of the replacement rate of RFP on the concentration of free Cl^−^ ions in a sodium chloride solution.

Figure 4 presents the concentration distribution of free Cl^−^ ions in the concrete following 240 days of immersion in a sodium chloride solution. Apparently, after 240 days of soaking, the final erosion depth of free Cl^−^ ions lies in the range of 15–20 mm. Moreover, as the replacement rate of RFP increases, the content of free Cl^−^ ions at the same depth also rises. When the replacement rate is 10%, the free Cl^−^ ion content at each depth is nearly identical to that in the control concrete. This suggests that a small quantity of RFP has a negligible impact on the Cl^−^ ion erosion of concrete. In the case of concrete with a 20% RFP replacement rate, the Cl^−^ ion concentration in the surface layer is slightly higher than that in the control concrete, whereas the concentration in the deep layer is lower. Thus, to maximize the utilization of RFP without significantly diminishing the concrete’s durability against Cl^−^ ion erosion, the optimal RFP replacement rate should be 20%.

### 3.2. Distribution of Cl^−^ Ion of Concrete in Brine Solution of Salt Lake

**Figure 5:**
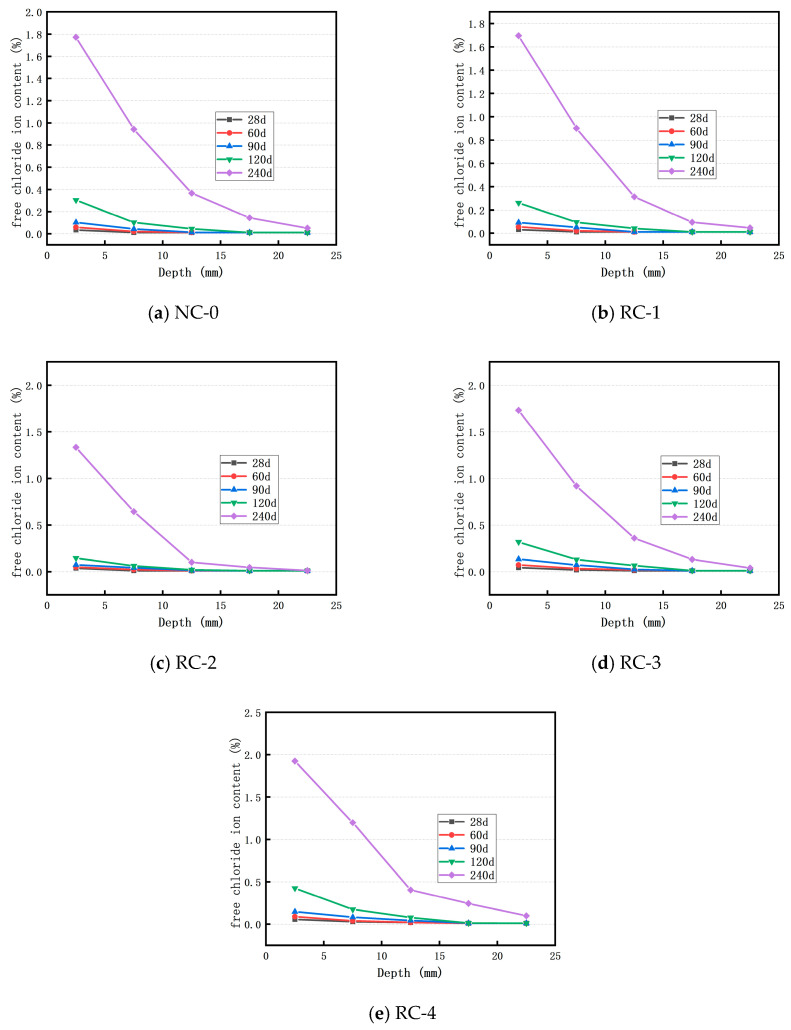
Distribution of the free Cl^−^ ion content of concrete in the Salt Lake brine solution.

Figure 5 depicts the distribution of the free Cl^−^ ion content at different depths within the control concrete and the concrete incorporating 10%, 20%, 30%, and 40% recycled fine powder (RFP) after being immersed in Salt Lake brine with a Cl^−^ ion concentration of 200 g/L for 28 days, 60 days, 90 days, 120 days, and 240 days, respectively. It can be observed that the erosion behavior in the brine is analogous to that in the sodium chloride solution. Specifically, for both the control concrete and the RFP-containing concrete, the Cl^−^ ion concentration and diffusion depth at the same position increase as the soaking time lengthens. However, there is a difference. The free Cl^−^ ion concentration at each depth does not exhibit a significant change prior to 90 days of soaking in the Salt Lake brine. When the soaking time reaches 240 days, the free Cl^−^ ion concentration at the same depth in the RFP-containing concrete surges abruptly. This is because in the early stage of erosion, the main ions in the brine are Cl^−^, SO4^2−^, Na^+^, and Mg^2+^, and the mutual inhibition among various ions leads to fewer Cl^−^ ions entering the concrete in the initial erosion stage. Meanwhile, sulfate-like corrosion damage occurs inside the concrete, causing expansion cracking of the concrete [31]. At this point, a large number of Cl^−^ ions penetrate into the interior of the concrete, resulting in a rapid increase in the free Cl^−^ ion content within the concrete.

**Figure 6:**
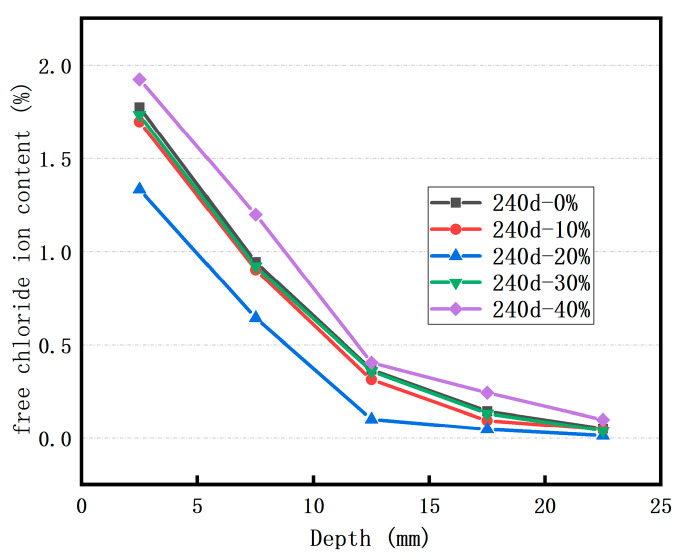
Effect of the replacement rate of RFP on the concentration of free Cl^−^ ions in the Salt Lake brine solution.

Figure 6 presents the distribution of the free Cl^−^ ion concentration in the concrete after 240 days of immersion in Salt Lake brine. Obviously, following 240 days of soaking, the final erosion depth of free chloride ions ranges from 20 to 25 mm. The Cl^−^ ion concentration in the concrete with 10% and 30% RFP is comparable to that in the control concrete at each depth. Notably, the Cl^−^ ion concentration at each depth in the concrete with 20% RFP is lower than that in the control concrete. This indicates that the concrete with a 20% RFP content demonstrates the best resistance to Cl^−^ ion erosion during brine immersion.

### 3.3. Effect of Soaking Time on Diffusion Coefficient D_f_ of Free Cl^−^ Ion

The free chloride diffusion coefficient (D_f_) of each group of samples was obtained by means of fitting regression analysis based on Fick’s second law and Origin software (9.5.1.195). Considering the boundary conditions *C*(0,*t*) = *C_s_*, *C*(*∞*,*t*) = *C*_0_, and the initial condition *C*(*x*,0) = *C*_0_, the one-dimensional mathematical solution is as follows:

(1) $$C\left(x,\;t\right)=C_{0}+(C_{s}-C_{0})\left[1-erf\left(\frac{x}{2\sqrt{Dt}}\right)\right]$$

(2) $$\mathrm{Error}\;\mathrm{function};\;\mathrm{erf}\left(z\right)=\frac{2}{\pi }\int_{0}^{z}e^{-u^{2}}dx$$

where *C*(*x*,*t*) is the Cl^−^ content in the concrete at x depth at time t (the percentage of Cl^−^ mass in the total mass of concrete), *C*_0_ is the initial Cl^−^ concentration in the concrete, *C_s_* is the Cl^−^ content on the concrete surface, *D* is the diffusion coefficient of Cl^−^ ions (mm^2^·s^−1^), *x* is the depth from the concrete surface (mm), and *t* is the exposure time (s) [26].

According to the C_f_ values at different depths in the concrete measured from the test, and in accordance with Formula (1), the free Cl^−^ diffusion coefficient (D_f_) of each group of specimens is obtained through fitting and regression using the Origin software.

**Figure 7:**
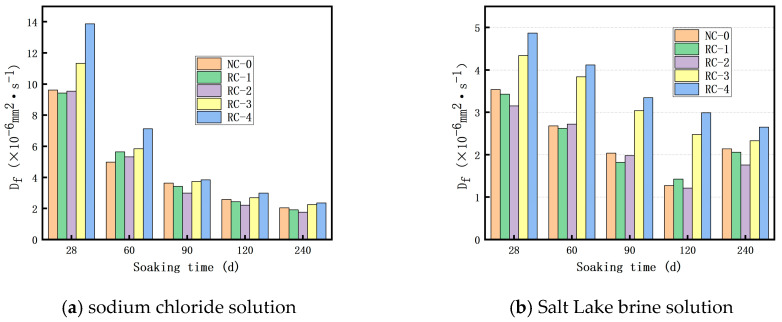
The relationship between soaking time and D_f_.

Figure 7 presents column charts of the free chloride ion diffusion coefficient D_f_ of concrete in a sodium chloride solution and a Salt Lake brine solution for soaking times of 28 d, 60 d, 90 d, 120 d, and 240 d. Apparently, the free Cl^−^ ion diffusion coefficient of concrete in both solutions decreases as the soaking time increases. In Figure 7a, at the early stage of soaking, the free Cl^−^ ion diffusion coefficient (D_f_) of concrete with 30% and 40% RFP is significantly higher than that of the control concrete. However, as the soaking time extends, at the middle stage (120 days of soaking), due to the secondary hydration of RFP, it plays a role in filling the internal pore structure of the concrete. Consequently, the internal structure of the concrete becomes denser, and the free Cl^−^ ion diffusion coefficient (D_f_) of the concrete approaches that of the control concrete. When soaked for 240 days, the secondary hydration effect of RFP is more pronounced, and the free Cl^−^ ion diffusion coefficient (D_f_) of the concrete is even closer to that of the control concrete. The free chloride diffusion coefficient (D_f_) of concrete with 10% and 20% RFP is lower than that of the control concrete after 90 days of soaking. It can be inferred that using RFP as a supplementary cementitious material can enhance the resistance of concrete to chloride ion permeability [32], yet it requires a certain period for the concrete to fully undergo rehydration. Figure 7b shows the free Cl^−^ ion diffusion coefficient of concrete soaked in the Salt Lake brine solution for 28, 60, 90, 120, and 240 days. It can be observed that the diffusion coefficient of free Cl^−^ ions in concrete gradually decreases with the extension of soaking time up to 120 days. However, after 240 days of soaking, the diffusion coefficient increases compared to that at 120 days, and the diffusion coefficients of free Cl^−^ ions in concrete with different mix ratios are similar. This test indicates that the ability of concrete to resist Cl^−^ ion diffusion in the Salt Lake brine solution environment over a long term period will decline, and the internal structure may be damaged.

As can be seen from Figure 7a, for the RFP concrete immersed in the sodium chloride solution, in the early stage of immersion, the free chloride ion diffusion coefficients of the RFP concrete with replacement rates of 20% and 30% are higher than those of the ordinary concrete. However, with the extension of the immersion time, in the middle stage of immersion, due to the secondary hydration of the RFP, the internal pore structure of the concrete is improved, and the inside of the concrete becomes denser. The D_f_ of the recycled fine powder concrete gradually approaches that of the ordinary concrete. When the immersion time reaches 90 days, the secondary hydration of the RFP is more obvious, and the D_f_ of the RFP concrete is lower than that of the concrete without RFP. This means that after the recycled fine powder partially replaces the cement, the chloride ion penetration resistance of the concrete is improved.

As can be seen from Figure 7b, for the RFP concrete immersed in the Salt Lake brine solution, in the early stage, there is not much difference in the free chloride ion diffusion coefficient between it and the ordinary concrete. This is mainly because there are a large number of sulfate ions in the Salt Lake brine solution, and in the early stage of erosion, sulfate ions and chloride ions are in a competitive relationship. The presence of sulfate ions will occupy part of the ion channels, reducing the chances of chloride ions entering, while at the same time decreasing the opportunities for chloride ions to combine with the hydration products of the concrete. However, as the immersion time increases and the concentration of chloride ions increases, the inhibitory effect gradually decreases. When it exceeds a critical value, the sulfate ions will promote the diffusion of chloride ions in the concrete. At the same time, the erosion of sulfate will also generate expansive substances such as gypsum inside the concrete, leading to the formation of large cracks and pores in the concrete, which makes the diffusion of chloride ions easier [33,34]. Therefore, it is shown that at 240 days, the free chloride ion diffusion coefficient of the concrete immersed in the Salt Lake brine solution is higher than that of the concrete immersed in the sodium chloride solution.

### 3.4. Comparative Analysis of Cl^−^ Ion Diffusion Properties After Soaking in Two Kinds of Salt Solution

#### 3.4.1. Cl^−^ Ion Erosion Concentration

**Figure 8:**
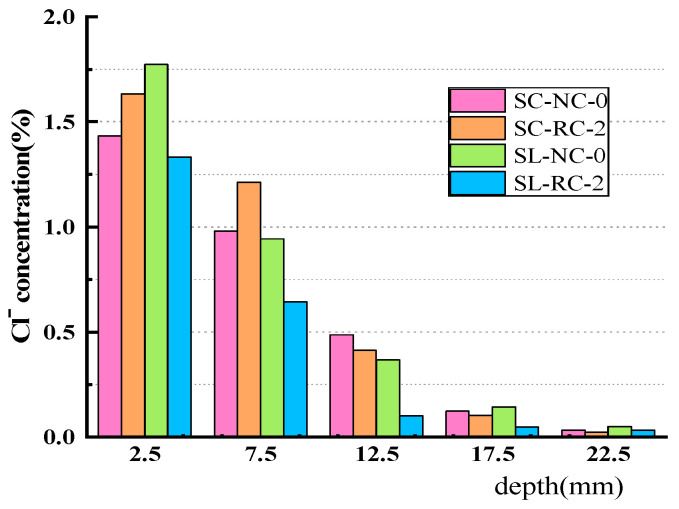
Comparison of Cl^−^ concentration in concrete between the sodium chloride solution and Salt Lake brine solution.

Figure 8 shows the corresponding concentrations of Cl^−^ ion diffusion at different depths of concrete soaked in the sodium chloride solution and the Salt Lake brine for 240 days, respectively (SC represents the sodium chloride solution, while SL represents the Salt Lake brine solution). It can be seen that after soaking for 240 days, the Cl^−^ ion diffusion depth in the sodium chloride solution and brine with the same dosage is the same. The diffusion depth of NC-0 is greater than that of RC-2. These results show that a proper amount of RFP can prevent the diffusion of free Cl^−^ ions in the solution.

#### 3.4.2. Free Cl^−^ Ion Diffusion Coefficient D_f_

**Figure 9:**
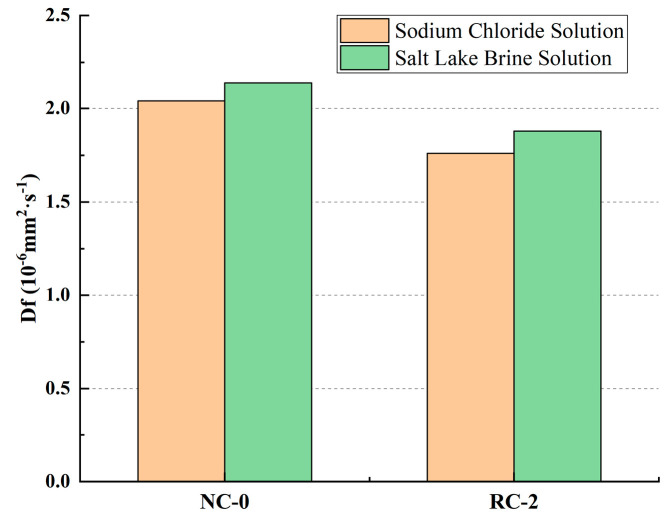
Comparison of the diffusion coefficient D_f_ after a soak time of 240 d.

Figure 9 shows the Cl^−^ ion diffusion coefficient D_f_ of concrete after soaking in the sodium chloride solution and Salt Lake brine for 240 days. It can be seen that after soaking for 240 days, the free Cl^−^ ion diffusion coefficients D_f_ of NC-0 and RC-2 soaked in the sodium chloride solution are 2.04 × 10^−6^ mm^2^·s^−1^ and 1.76 × 10^−6^ mm^2^·s^−1^, respectively, and those soaked in the Salt Lake brine are 2.14 × 10^−6^ mm^2^·s^−1^ and 1.88 × 10^−6^ mm^2^·s^−1^, respectively. Compared with the erosion in the sodium chloride solution, Salt Lake brine causes greater damage to the concrete.

### 3.5. SEM Analysis

**Figure 10:**
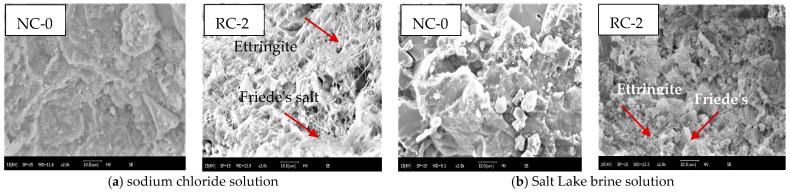
SEM of NC-0 and RC-2.

Figure 10 presents the SEM images of NC-0 and RC-2 after brine soaking for 120 days. As can be seen from Figure 10a, when the immersion time in the sodium chloride solution reaches 120 days, the internal structure of the concrete NC-0 is relatively dense. However, in the concrete RC-2 with 20% RFP added, a large number of salt crystals and acicular ettringite are present inside, and there are also more internal pores. As shown in Figure 10b, after NC-0 has been immersed in the Salt Lake brine solution for 120 days, salt crystalline substances begin to form on its surface, and the number of pores increases. In RC-2, salt crystals and ettringite also start to form. However, compared with the situation in the sodium chloride solution, the amount of salt crystalline substances generated is lower, and the structure is relatively dense. This indicates that at this time, the destructive effect of sulfate crystallization is not yet obvious.

### 3.6. XRD Analysis

**Figure 11:**
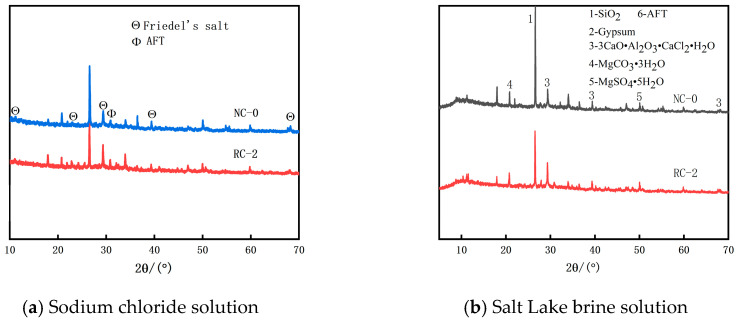
XRD of NC-0 and RC-2.

Figure 11 presents the X-ray diffraction (XRD) patterns of NC-0 and RC-2 after being soaked in the salt solutions for 240 days. Under both mix ratios, Friedel’s salt (3CaO·Al_2_O_3_·CaCl_2_·H_2_O) is generated, and the peak value of the concrete with 20% RFP is higher than that of ordinary concrete. This indicates that the RFP enhances both the physical adsorption and chemical reaction capabilities of chlorides, and free Cl^−^ is more prone to forming Friedel’s salt that fills the pores in the concrete [14]. As a result, the diffusion rate of Cl^−^ in the concrete is reduced, enhancing the concrete’s resistance to chloride salt erosion. Meanwhile, in the Salt Lake solution, both MgSO_4_ salt crystals and gypsum were found in NC-0 and RC-2. This verified that sulfate erosion damage had occurred at this stage.

## 4. Discussion

This study confirms the significant impact of RFP on the chloride ion erosion resistance of concrete, and the experimental results are consistent with previous research, reinforcing the reliability of exploring the chloride ion penetration resistance of recycled fine powder concrete when it is immersed in a sodium chloride solution and a Salt Lake brine environment for a long time under natural conditions, which can provide a certain theoretical basis for practical engineering projects. However, due to time limitations and various uncertain factors, the test results inevitably have certain limitations.

First, the results of this study indicate that when the soaking time reaches 240 days, the diffusion rate of chloride ions in both solutions is very limited. However, if the soaking time is further extended, whether there will be significant differences in the diffusion behavior of chloride ions in these two solution environments is something that this current study has not been able to address. Further experimental research and discussion in the future are needed.

In addition, this study only used RFP from a single source. Since the properties of RFP can vary significantly depending on its source, if RFP made from different construction waste materials such as waste bricks is used, the experimental results may differ. At the same time, RFP from a single source has a relatively uniform particle size and morphology. When it is incorporated into concrete, due to the limited activity of the recycled fine powder, its ability to improve the durability of concrete, such as its resistance to chloride ion erosion, is also rather limited. Therefore, in the future, consideration should be given to the combined use of recycled fine powder with mineral admixtures of different fineness, such as fly ash and silica fume, to improve the durability of concrete.

## 5. Conclusions

In this study, the effects of the substitution rate of RFP, type of salt solution, and soaking time on the free chloride ion diffusion performance in concrete were investigated. The following conclusions are obtained:

- The Cl^−^ ion concentration in concrete at the same depth increases as the soaking time lengthens. Incorporating an appropriate amount of RFP into concrete can enhance the concrete’s resistance to Cl^−^ ion penetration. When the RFP replacement rate is 20%, the concrete demonstrates good resistance to Cl^−^ ion erosion in both the sodium chloride solution and the Salt Lake brine solution.
- In both the sodium chloride solution and the Salt Lake brine solution, as the soaking time increases, the free chloride ion diffusion coefficient (D_f_) of the RFP concrete shows a downward trend, indicating that after the RFP concrete is fully cured, the recycled fine powder undergoes secondary hydration, which improves the compactness of the concrete and enhances its durability. The hydration products of the RFP concrete are rich in mineral components such as tricalcium aluminate hydrate, which can react with the free Cl^−^ in the concrete to form Friedel’s salt, solidifying part of the Cl^−^, thus effectively delaying the diffusion rate of Cl^−^ in the concrete.
- Compared with erosion in the sodium chloride solution, after soaking in Salt Lake brine for 240 days, the Cl^−^ ion diffusion coefficients D_f_ of NC-0 and RC-2 are 2.14 × 10^−6^ mm^2^·s^−1^ and 1.88 × 10^−6^ mm^2^·s^−1^, respectively, increasing by 0.1 × 10^−6^ mm^2^·s^−1^ and 0.12 × 10^−6^ mm^2^·s^−1^ compared with the erosion in the sodium chloride solution. The results show that the damage degree in the Salt Lake solution is stronger than in the sodium chloride solution at the latter age. This indicates that as the soaking time increases, the inhibitory effect of sulfate ions in the Salt Lake brine on the penetration of chloride ions gradually disappears. Sulfate erosion leads to the generation of a large amount of volume-expanding substances, increasing the number of pores in the concrete, which makes the penetration of chloride ions easier.

## Figures and Tables

**Table 1:** Chemical composition of raw materials.

Material	CaO	SiO_2_	Al_2_O_3_	Fe_2_O_3_	MgO	CO_2_	SO_3_
Cement	67.7	12.0	3.02	6.77	0.09	6.12	2.37
RFP	34.5	25.8	4.44	6.04	0.15	25.80	0.44

**Table 2:** Physical properties of RFP.

Material	Fineness (45 μm)/%	Bulk Density/(kg·m^−3^)	Apparent Density/(kg·m^−3^)	Water Demand Ratio/(%)	Specific Surface Area/(m^2^/kg)	Activity Index/(%)
RFP	30.78	2483	945	116	467	0.73

**Table 3:** Physical properties of aggregates.

Materials	Bulk Density /kg·m^−3^	Apparent Density /kg·m^−3^	Clay Content/%	Water Content/%
Coarse aggregate	1510	2673	0.33	0.38
Fine aggregate	1501	2673	1.14	1.15

**Table 4:** The main ionic concentrations in Salt Lake brine, g/L.

	Cl^−^	Mg^2+^	SO4^2−^	Ca^+^	K^+^	Na^+^	Li^+^
Salt Lake brine	201	153	104	0.35	79	12	0.24

**Table 5:** Design of concrete mixing proportions.

NO.	Replacement Rate/%	W/C	Amount of Materials/(kg/m^3^)			
			Cement	RFP	Gravel	Sand
NC-0	0	0.45	455	0	1032	595
RC-1	10	0.45	410	46	1032	595
RC-2	20	0.45	364	91	1032	595
RC-3	30	0.45	319	137	1032	595
RC-4	40	0.45	273	182	1032	595

## Data Availability

The original contributions presented in this study are included in the article; further inquiries can be directed to the corresponding authors.

## Abbreviations

The following abbreviations are used in this manuscript:

RFP	recycled fine powders
D_f_	the free chloride ion diffusion coefficient
Friedel’s salt	3CaO·Al_2_O_3_·CaCl_2_·10H_2_O
C_3_A	tricalcium aluminate hydrate
C_f_	the mass percentage of Cl^−^ content relative to the mass of concrete
